# Multifunctional Optoelectronic Synapses Based on Arrayed MoS_2_ Monolayers Emulating Human Association Memory

**DOI:** 10.1002/advs.202300120

**Published:** 2023-04-14

**Authors:** Ming Huang, Wajid Ali, Liuli Yang, Jianhua Huang, Chengdong Yao, Yunfei Xie, Ronghuan Sun, Chenguang Zhu, Yike Tan, Xiao Liu, Shengman Li, Ziwei Li, Anlian Pan

**Affiliations:** ^1^ Key Laboratory for Micro‐Nano Physics and Technology of Hunan Province Hunan Institute of Optoelectronic Integration College of Materials Science and Engineering Hunan University Changsha Hunan 410082 P. R. China

**Keywords:** artificial synapse, association memory, MoS_2_, optoelectronic device

## Abstract

Optoelectronic synaptic devices integrating light‐perception and signal‐storage functions hold great potential in neuromorphic computing for visual information processing, as well as complex brain‐like learning, memorizing, and reasoning. Herein, the successful growth of MoS_2_ monolayer arrays assisted by gold nanorods guided precursor nucleation is demonstrated. Optical, spectral, and morphology characterizations of MoS_2_ prove that arrayed flakes are homogeneous monolayers, and they are further fabricated as optoelectronic devices showing featured photocurrent loops and stable optical responses. Typical synaptic behaviors of photo‐induced short‐term potentiation, long‐term potentiation, and paired pulse facilitation are recorded under different light stimulations of 450, 532, and 633 nm lasers at various excitation powers. A visual sensing system consisting of 5 × 6 pixels is constructed to simulate the light‐sensing image mapped by forgetting curves in real time. Moreover, the system presents the ability of utilizing associated images to restore vague and incomplete memories, which successfully mimics human intelligent behaviors of association memory and logical reasoning. The work emulates the brain‐like artificial intelligence using arrayed 2D semiconductors, which paves an avenue to achieve smart retina and complex brain‐like system.

## Graphical abstract

The growth of MoS_2_ monolayer arrays assisted by gold nanorods is demonstrated. Arrayed devices present the ability of utilizing associated images to restore vague and incomplete memories, which successfully mimics human intelligent behaviors. The work emulates the brain‐like artificial intelligence using arrayed 2D semiconductors, which paves an avenue to achieve smart retina and complex brain‐like system.

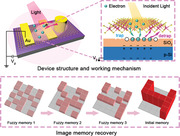

## 1 Introduction

The human brain system is born with the abilities of learning and memorizing, which are arising from a series of complex biological reactions, including light harvesting in photosensory cell, signal transduction between neural and synapse, and pre‐processing of image information before the brain conducting more physiological reactions.^[^ ^1^ ^]^ The rigorous development of artificial intelligence promotes the research of designing biomimetic optoelectronic systems to imitate functions of human retina and brain. Recently, optoelectronic artificial synapses based on two‐dimensional (2D) semiconductors have been explored widely to convert photons into carriers, as well as to process and remember signals realizing versatile brain‐like behaviors in image recognition,^[^ ^2^, ^3^ ^]^ control of mechanic robots,^[^ ^4^ ^]^ and learning assistance.^[^ ^5^, ^6^ ^]^ Optoelectronic synapses with this concept can break through the bottleneck of information computing in the framework of von Neumann, such as traditional charge‐coupled devices (CCD) or complementary metal–oxide silicon (CMOS) imagers.^[^ ^7^, ^8^ ^]^ However, limited by the development of fabrication technique, artificial visual systems presenting intelligent behaviors have rarely been reported, like association memory and logical reasoning of human.

Molybdenum disulfide (MoS_2_) as a typical van der Waals 2D semiconductor, has been widely reported to work as the key fundamental material in optoelectronic synapses.^[^ ^9^, ^10^ ^]^ The basic synaptic characteristics of excitatory postsynaptic current (EPSC), photo‐induced short‐term potentiation (STP) and long‐term potentiation (LTP), paired pulse facilitation (PPF), and spike time dependent plasticity (STDP), have been systematically studied in MoS_2_ synaptic devices,^[^ ^11^, ^12^ ^]^ and their device performances of various MoS_2_ flakes are compared among chemical vapor deposition (CVD) growth,^[^ ^13^ ^]^ mechanical exfoliation,^[^ ^14^ ^]^ and MoS_2_ in vacuum conditions. Besides, MoS_2_ monolayers are designed to integrate with graphene or hexagonal boron nitride (*h*‐BN) layer exhibiting efficient charge trap and release for the application of neuromorphic engineering.^[^ ^15^, ^16^ ^]^ Moreover, MoS_2_‐based heterostructures with organic molecules,^[^ ^17^ ^]^ quantum dots,^[^ ^18^, ^19^ ^]^ and plasmonic nanoparticles^[^ ^20^ ^]^ have been explored to present complicated functions and excellent performances in neuromorphic learning and logical memory. Although, these efforts have been devoted to present the ability of biomimetic learning and memory in 2D semiconductor devices,^[^ ^21^, ^22^, ^23^ ^]^ most works only report the device performances based on a single optoelectronic synapse with limited optoelectronic performances,^[^ ^24^, ^25^, ^26^ ^]^ and it still faces great challenges in fabricating arrayed devices for the practical application of retina‐like and brain‐like synapses.^[^ ^27^, ^28^ ^]^

Here, we demonstrate the controllable growth of MoS_2_ arrays guided by periodic gold nanorods. Photoluminescence (PL) and Raman spectral characterizations confirm that our grown MoS_2_ flakes are homogeneous monolayers in high quality. Benefitting from charge trap and release of plasmon‐induced electrons at the interface of MoS_2_/SiO_2_, high and low resistance states have been measured in photocurrent loops of photodetectors, and arrayed devices keep stable and repeatable photoresponses after dozens of cycles. Moreover, synaptic behaviors of STP, LTP, and PPF in devices have been systematically investigated under pulsed light excitations with various power densities and lasing wavelengths. It is found that the higher pulsed frequency, the stronger power density and the nearer bandgap excitation can stimulate more remarkable optoelectronic synaptic behaviors of fast learning and long‐time memory. We further explore the device functions in restoring forgotten images by connecting and comparing association memories of reference images, which exhibits the potential abilities in mimicking human's scientific mnemonics of association memory. Our work promotes the development of brain‐like integrated devices based on arrayed 2D semiconductors, which opens up an avenue toward complex brain‐like functions in association, memory, and logical reasoning.

## 2 Results and Discussions

**Figure 1:**
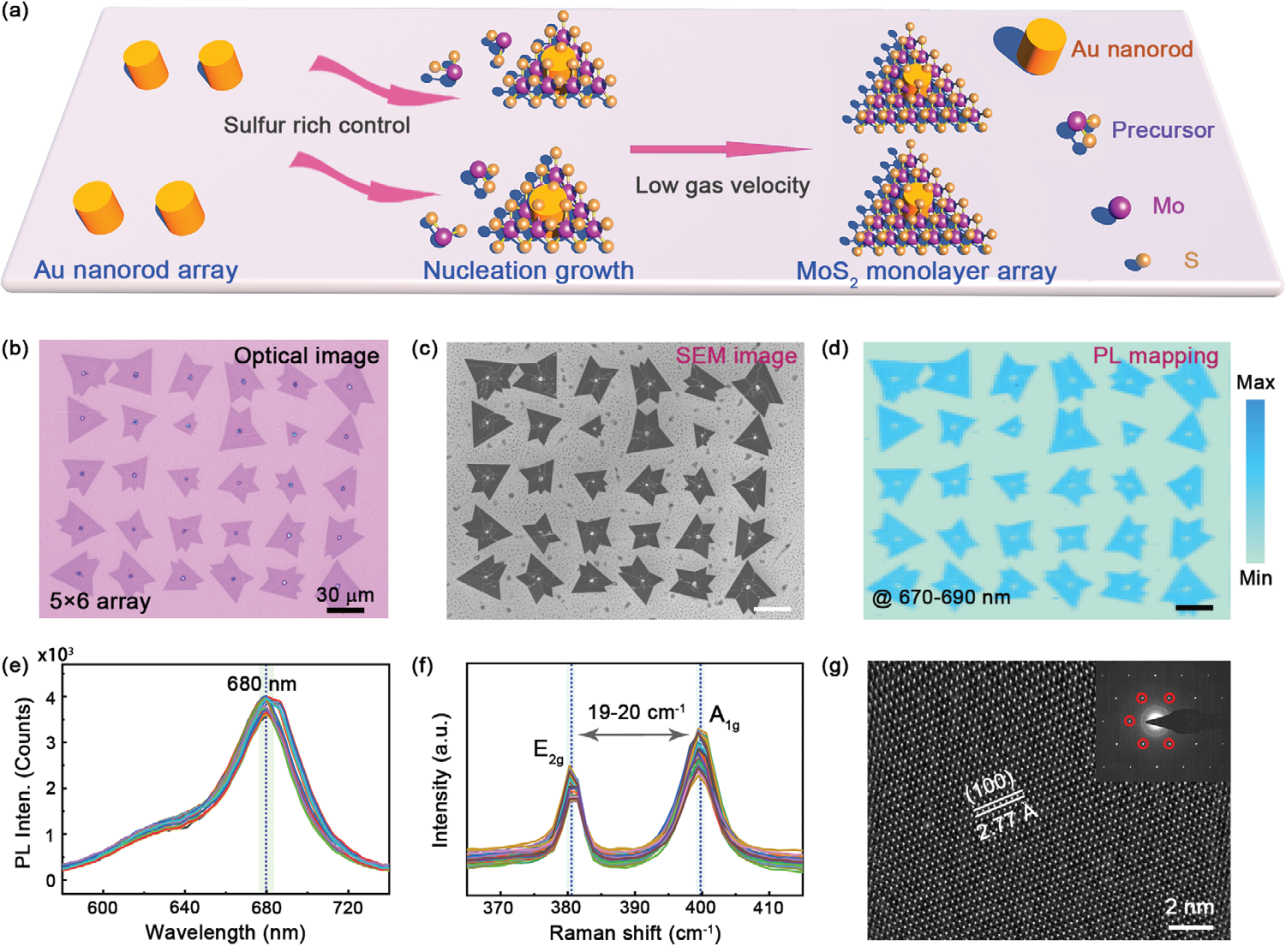
Schematics of growth, morphology, and spectral characteristics. a) Schematic view of CVD growth of arrayed MoS_2_ monolayers guided by Au nanorods. The control of sulfur‐rich component in precursors and low gas velocity help to realize the monolayer growth of MoS_2_. b) Optical image of 5×6 array of MoS_2_ monolayers grown at the location of Au nanorods. c) SEM image of MoS_2_ monolayer arrays showing clear structure morphology from the contrast. d) Mapping image of integrated PL intensity of MoS_2_ monolayer arrays at 670–690 nm. Scale bars are 30 µm. e) PL spectra detected from thirty monolayers exhibiting stable luminescence peaks and uniform intensities. f) Raman spectra detected from thirty monolayers showing that the difference of Raman shifts is in the range of 19–20 cm^−1^. g) TEM image of MoS_2_ monolayer presenting clear hexagonal lattice with a spacing distance of 0.27 nm. The scale bar is 2 nm. The inset shows the corresponding FFT pattern.

Arrayed MoS_2_ monolayers and their devices can be achieved in a series of preparation processes. **Figure** **1**a shows the schematic view of CVD growth of MoS_2_ arrays. First, gold nanorod arrays on SiO_2_/Si substrate were designed and fabricated using direct laser‐writing photolithography and film deposition (Supporting Information S1). Then, the substrate with nanorod arrays was put into the tube of a furnace to guide the precursor accumulation and nucleation. Nanorods can induce both space and energy barriers to adsorb precursor molecules, which provide location templates leading the ordered growth of MoS_2_ flakes.^[^ ^29^ ^]^ Besides, Au element in nanorods can be partially doped into MoS_2_ layers during the high temperature evaporation, resulting in the improved optoelectronic properties of semiconductors.^[^ ^20^, ^30^ ^]^ Moreover, nanorods behave strong surface plasmon resonance under the light excitation, generating huge field enhancement and hot electron doping overlapping the whole region of MoS_2_ layers.^[^ ^31^ ^]^ Finally, under the precise control of source ratio and gas velocity, homogeneous MoS_2_ monolayers were successfully grown forming arrayed structures. 2×2, 3×2, and 5×6 arrays of MoS_2_ monolayers were successfully prepared (Supporting Information S2).

Although, this strategy induces few additional steps compared with conventional wafer‐scale CVD growth followed by standard lithography technology, which still has inherent advantages in two aspects: one is the precise guidance of monolayer growth of 2D semiconductors, which is due to the catalytic induction of molecule nucleation by Au‐nanorods; the other is the significant enhancement of light‐matter interactions of 2D semiconductors, where Au‐nanorod induced plasmonic field enhancement and electron doping can improve the photoelectric conversion efficiency of devices in limited physical space.

Figure 1b shows the optical image of 5×6 array of MoS_2_ flakes, where MoS_2_ flakes are always centered around gold nanorods, and the thickness uniformity of MoS_2_ can be obviously observed from the optical contrast. The diameter and the height of the nanorod are 1 µm and 60 nm, respectively. The corresponding scanning electron microscope (SEM) image is shown in Figure 1c, where detailed surface morphology and grain boundary can be further characterized. In Figure 1d, PL mapping image presents the homogeneous distribution of spectral intensity collected from 670 to 690 nm. Scale bars in these patterns are 30 µm. PL and Raman spectra of MoS_2_ are plotted in Figure 1e,f, respectively, where each spectrum is measured from each MoS_2_ flake at random locations. The spectral shape and intensity are highly similar, and the difference of two featured Raman peaks is serious in the range of 19–20 cm^−1^, which prove that grown MoS_2_ flakes are monolayers. To characterize the lattice quality of materials, MoS_2_ monolayers are transferred on Cu mesh to obtain the ultrahigh resolution image and fast Fourier transform (FFT) pattern in Figure 1g. The lattice structures exhibit clear hexagonal atomic structure, and the lattice spacing is determined to be 0.27 nm, which consists well with previous reports. All spectral and structure characterizations confirm that the controllable growth of arrayed MoS_2_ monolayers has been realized in our work.^[^ ^32^, ^33^ ^]^

**Figure 2:**
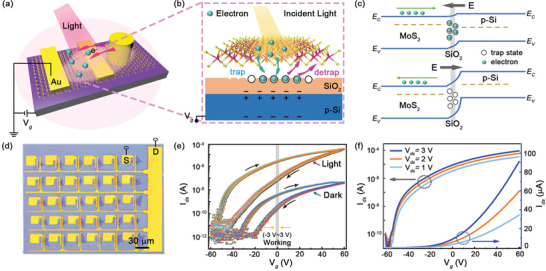
Fabrication of arrayed devices and *I*–*V* measurements. a) Schematic of a MoS_2_ synaptic transistor with a gold‐nanorod working under the light illumination. A large amount of plasmonic doping electrons can be injected into MoS_2_ monolayers for the contribution of efficient photocurrent. b) The enlarged view of electrons trapped or released at the interface of MoS_2_/SiO_2_ revealing the working mechanism of synaptic behaviors. c) Energy diagram of heterostructures and their electron migrations at both forward and reverse electric fields. d) Optical image of arrayed electrodes fabricated on MoS_2_ monolayers. Scale bar is 30 µm. e) Transfer characteristic curves of 30 devices at *V*_ds_ = 1 V in dark and under light illumination (wavelength of 532 nm, power of 0.12 mW cm^−2^). Ideal working voltage ranges from −3 to 3 V (gray region). f) *I*_ds_–*V*_g_ curves at various drain biases of 1, 2, and 3 V.

**Figure** **2**a presents the schematic image of a MoS_2_ optoelectronic device working under light illuminations, which is constructed with a neighboring Au‐nanorod, as well as two terminals controlled at voltages of *V*_ds_ and *V*_g_. Although, the Au‐nanorod is not located at the channel region, light‐excited plasmon‐electrons doped from Au‐nanorod can also inject into the MoS_2_ monolayer spreading the whole region of semiconductor flake. The generation mechanism of device photocurrent is illustrated in Figure 2b, that light‐created electrons and holes can be separated and harvested at the interface of MoS_2_/SiO_2_. Carrier transfer and transport behaviors can be modulated by tuning *V*_ds_ and *V*_g_, respectively.^[^ ^34^ ^]^ The sensitive trapping and the slow release processes of electron show advantages in potential application of optoelectronic synapse. Moreover, the physical mechanism of charge trap at the interface on the resistive switching under an external electric field has been illustrated in Figure 2c. When the device is biased with the forward electric field (arrow facing left), the energy barrier gets decreased. Electrons are trapped by the interfacial trap sites, and then MoS_2_ semiconductor becomes less conductive, resetting the device into the high resistance state (HRS). While for the device is biased with a reverse electric field (arrow facing right), the energy barrier is increased. Electrons tend to detrap and migrate to MoS_2_ monolayer, and the device works in the low resistance state (LRS). The switch between LRS and HRS is highly related to the trap/release of electrons at the interface.^[^ ^35^ ^]^ Figure 2d shows the optical image of arrayed devices with a scale bar of 30 µm. From the optical image, arrayed source, and drain electrodes are precisely fabricated on MoS_2_ monolayers, where drain electrodes of arrayed devices are connected and gated together.

Figure 2e presents the transfer characteristic curves of 30 MoS_2_ devices exhibiting the n‐type conductive behaviors and a maximum on/off ratio of 10^4^ in dark. Besides, the arrayed devices exhibit the mnemonic behaviors during a sweeping loop of the bias voltage changing from −60 via +60 V to −60V, where the resistance changes from HRS to LRS yielding a current hysteresis. When devices are illuminated by 532 nm laser, photogenerated carriers can cause a negative shift of threshold voltage and an increase of drain current. Massive photogenerated carriers are generated by plasmonic resonance and material absorption, which can inject into MoS_2_ monolayer causing the serious n‐type doping and a negative shift of threshold voltage. The on/off ratio of photocurrent becomes increased as large as 10^6^ due to the light illumination, and the mnemonic behavior becomes more remarkable and obvious. Balanced with the large mnemonic ability and high reliability of the device, the ideal working voltages should be in the range of −3 to 3 V, and these voltages are selected as control voltages to study optoelectronic performances of devices systematically. Figure 2f shows transfer characteristic curves of a typical device at various *V*_ds_ of 1, 2, and 3 V. The electron mobility is calculated as 0.62 cm^2^ V^−1^ s^−1^ (L = 15 µm, W = 4 µm, *V*_ds_ = 1 V), which is quite good compared with other MoS_2_ devices due to the realization of ohmic contact between electrode and MoS_2_. Besides, statistic results of output characteristic curves of 30 MoS_2_ devices at *V*_g_ = 0 V in dark confirm the uniform optoelectronic performances in all devices (Supporting Information S3).

**Figure 3:**
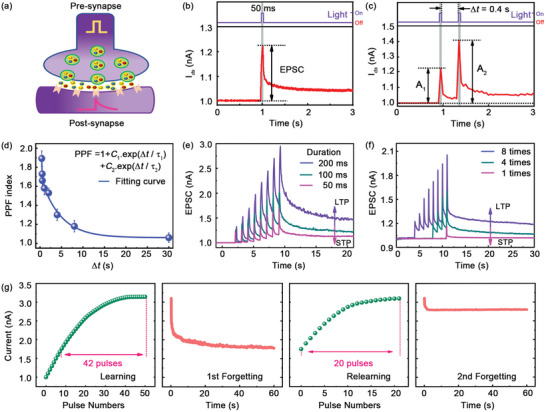
Synaptic plasticity of MoS_2_ optoelectronic device. a) Schematic illustration of a biological synapse with pre‐synapse stimuli, signal transduction, and postsynapse response. b) An EPSC of a synaptic transistor triggered by an optical spike. c) EPSC of a synaptic transistor triggered by a pair of optical spikes with the duration time of 0.4 s. d) The PPF index plotted as a function of inter‐spike interval (Δ*t*) and fitted by exponential curves. STM‐to‐LTM transition induced by increasing the duration time e) and the number of pulsed light stimuli f). g) Measured “learning‐forgetting” behaviors emulated by pulsed light stimuli. All measurements were performed at *V*_ds_ = 1 V and *V*_g_ = −3 V. The wavelength, duration time, and intensity of all optical spikes are 532 nm, 50 ms, and 0.21 mW cm^−2^, respectively.

**Figure** **3**a shows the schematic view of working mechanism in a biological synapse, which consists of two terminals containing pre‐ and postsynaptic neurons. When a biological signal is created from the presynapse, it will be delivered along the neuron and transduced to the postsynaptic unidirectionally owing to the asymmetric biological structure and function.^[^ ^36^ ^]^ Pre‐ and postsynaptic neurons possess dendritic donors and receptors, respectively, which help to release and sense neurotransmitters, enabling the conveyance of signals in neural circuits to form an EPSC in postsynapse. The plasticity of synaptic EPSC is one of the significant features of human “learning” and “memorizing” processes.

MoS_2_ monolayer devices are systematically investigated to understand their fundamental synaptic behaviors. Figure 3b shows the single learning behavior of the device, where a photocurrent response of 1.2 nA is obtained, which is triggered by a short pulse (50 ms) under the light intensity of 0.21 mW cm^−2^. The total energy consumption including the optical and electric supply energies is calculated by

(1) $$E\;=V_{\mathrm{ds}}\;\times \int I_{\mathrm{ds}}\;d_{t}+V_{g}\times \int I_{g}\;d_{t}+P_{\mathrm{light}}\times A\times t$$

where *I*_ds_, *I*_g_, *t*, *V*_ds_, *V*_g_, *P*_light_, and *A* are the drain current, the gate current, the spike duration time, the drain voltage (1 V), the gate voltage (−3 V), the light intensity and the effective area of device, respectively.^[^ ^20^ ^]^ The estimated total energy consumption is about 66.5 pJ for a single light stimulus in Figure 3b, and the power consumption data of 30 different devices are similar (Supporting Information Table S1). The minimum energy consumption is obtained as 26.9 pJ with the pump fluence of 1.5 *µ*W cm^−2^ (Supporting Information S4), which is quite low compared with other optoelectronic synaptic transistors based on typical low‐dimensional semiconductors (Supporting Information Table S2). Besides, the learning‐forgetting‐relearning processes are performed under the irradiation of a pair of light pulses with an interval (Δ*t*) of 0.4 s, as shown in Figure 3c. The value of *A*_2_/*A*_1_ is defined as PPF, which represents a transient memory feature exhibiting the ability of transmitting temporary information in synapse.^[^ ^37^ ^]^ *A*_2_ and *A*_1_ are peak intensities of EPSCs for the first and the second pulse, respectively. The value of our device here is detected as 1.9, larger than 1, which indicates that the number of photon‐induced electrons are enhanced at the second pulse stimuli. This feature boosts a higher exciting photocurrent and a longer decay period to mimic “learning,” “memorizing,” and “forgetting” abilities for high‐performance optoelectronic synapse.

Figure 3d shows the PPF index as a function of inter‐spike interval (Δ*t*). The values of PPF rapidly decrease from 1.9, and then gradually approach the value of 1.0 as the inter‐spike interval increases. Interestingly, experimental results can be fitted well by a double‐exponential decay relation (the blue line)

(2) $$PPF\;=\;1+C_{1}\cdot \exp\left(-\frac{\Delta T}{\tau_{1}}\right)+C_{2}\cdot \exp\left(-\frac{\Delta T}{\tau_{2}}\right)$$

where *C*_1_ and *C*_2_ are the original facilitation magnitudes of the two phases, respectively. *τ*_1_ and *τ*_2_ are their corresponding characteristic relaxation times, and they are determined to be 87 and 4506 ms by fitting the experimental results, respectively. The time scales of the rapid (tens of milliseconds) and slow (thousands of milliseconds) phases are comparable to those of biological synapses.

The device behaviors of STP and LTP are further systematically investigated. Figure 3e shows the duration‐time dependent ESPC measurements. After eight times stimulus (532 nm) with the duration time of 50, 100, and 200 ms, current (*I*_ph_) of MoS_2_ device surges immediately, then decreases gradually and reaches their stable values at 22 s. The peak currents get stronger as duration time increases from 50 to 200 ms. In addition, device performances on the number of pulses are shown in Figure 3f. The photocurrent increases dramatically with repeated light pluses, then maintains a high value for a LTP process. The switch of working mode can be realized by controlling the number of stimuli and the duration‐time of pulsed light.

Meanwhile, typical “learning‐memorizing‐forgetting” behaviors of MoS_2_ device have been emulated using two sequences of successive light pulses with an interval of 50 ms, as shown in Figure 3g. The first grouped pulses (42 pulses) are shined on the device to imitate the first learning process of the human brain, where the synaptic photocurrent increases at first, and then appears to be saturated. This behavior corresponds to the phenomenon that human brain tends to be tiring after multiple repeated learning processes. Subsequently, the photocurrent appears a spontaneous decay to an intermediate level after removing the light stimuli, which consists with the human brain behaviors of losing memorized information after a period of time. In the second learning process, only 20 pulses are required to achieve the same current (synaptic weight) as that in the first learning process, which reflects that less time is needed for the relearning process. It can be associated with the situation that human beings usually learn faster in the second relearning process. Moreover, the decay of synaptic weight in the second forgetting process is weaker than that in the first forgetting process, analogous to human's the long‐time memory ability after repeated learning times. Although, the mechanism of electron detrapping in two forgetting processes is the same, the distribution and the density of holes have already been changed after the learning‐forgetting‐relearning process, that means, partial holes have been firmly occupied by doping electrons before the second forgetting process, and arranged distribution of reduced holes could cause the slow current decay of the second forgetting process.

**Figure 4:**
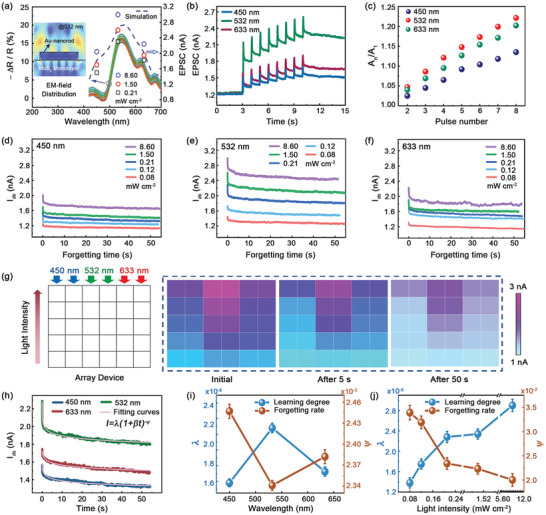
Photoresponsive characteristics of MoS_2_ devices under multiwavelength irradiations. a) Difference of reflection spectra of 30 MoS_2_ monolayers (left *y*‐axis) in experiments. Absorption spectra of Au‐nanorod on MoS_2_/SiO_2_/Si substrate from simulation (left *y*‐axis). Intensity variation of EPSC under 450, 532,  and 633 nm irradiations (right *y*‐axis). Inset is the electromagnetic field distribution of a gold‐nanorod on MoS_2_/SiO_2_/Si substrate. b) EPSC curves stimulated with three different wavelengths at the same power density of 1.50 mW cm^−2^. c) Pulse‐number‐dependent gain of *A*_n_/*A*_1_ under multiwavelength stimulations. Pump‐fluence dependent forgetting curves plotted at 450 nm d), 532 nm e), and 633 nm f). All intensities of light stimuli are detected at *V*_ds_ = 1 V and *V*_g_ = −1 V with an interval of 50 ms. g) Mimicry of human visual memory in an array of 5×6 synapses. Schematics of illuminated regions with various wavelengths and light intensities (left). Color maps of photocurrent intensity at the initial state, after 5 s and after 50 s (right). h) Current decay is well fitted by the Wickelgren's power law. i) Changes in forgetting rate *ψ* and learning degree *λ* at different pulse wavelengths. j) Changes in forgetting rate *ψ* and learning degree *λ* with various light intensities of 532 nm laser.

**Figure** **4**a shows the differential reflection spectra of MoS_2_ monolayers detected at random positions (left *y*‐axis), which exhibit consistent spectral shape and intensity. The dotted line shows the simulation result of absorption spectra of a gold‐nanorod on MoS_2_ monolayer (left *y*‐axis). The values of EPSC stimulated with different lasers and various pump fluences are also plotted in Figure 4a (right *y*‐axis), which agree well with the trend of simulated spectral line‐shape. The maximum EPSC values are measured under 532 nm excitation, which is close to the main absorption peak (around 540 nm) of plasmonic resonance.^[^ ^38^ ^]^ The generation mechanism of photocurrent mainly relies on Au‐nanorod induced plasmonic effect. Inset shows the simulation result of electromagnetic field distribution of a gold‐nanorod on MoS_2_/SiO_2_/Si substrate, where giant field enhancements are observed near the interface of nanorod and MoS_2_. Figure S5 (Supporting Information) provides the detailed information of wavelength‐dependent field enhancement, where the strongest and the weakest plasmonic resonances are observed at 532 and 450 nm, respectively. Usually, intenser plasmonic resonance can produce more “hot” electrons to dope the whole MoS_2_ monolayer, resulting in the wavelength‐sensitive detection of EPSC. Figure 4b shows the wavelength‐sensitive EPSC curves with eight light stimuli (1.50 mW cm^−2^), where the maximum values are detected under 532 nm excitation. *A*_n_ is peak intensity of EPSC for the nth pulse, and the value of *A*_n_/*A*_1_ represents the memorizing ability after multiple learning processes. Figure 4c shows the *A*_n_/*A*_1_ values as a function of pulse number. Under the stimulation of a certain wavelength, the *A*_n_/*A*_1_ value gets increased as the pulse number increases, and the highest values of *A*_n_/*A*_1_ are always measured under 532 nm irradiation.

Figure 4d–f compares the decay ability of EPSC curves after eight light stimuli under the excitation of three different lasers with various pump fluences, where the range of *y*‐axis is normalized from 0.9 to 3.5 nA (Supporting Information S6 and Table S3, Supporting Information). It can be obviously observed that the highest decay values of EPSC are always detected under 532 nm excitation. From above experimental results, it can be concluded that the device performance of MoS_2_ synapse can be optimized and improved by selecting a suitable excitation wavelength close to the absorption peak of material, or by increasing the pump fluence to generate more photo‐induced carriers.

Possessing optical information detecting, processing and memorizing capabilities, MoS_2_ optoelectronic synapses are suitable for mimicking human visual memory. Experimentally, 30 arrayed synapses are utilized to recognize and memorize the input images consisting of 5 × 6 pixels, and each was stimulated by eight light pulses (pulse width of 50 ms) with predefined laser and light intensity, followed by the decay measurement of EPSC for 50 s, as shown in Figure 4g. Color maps of photocurrent intensity present the measured results for an input image encoded by light wavelength and intensity at three states of initial state, decay after 5 s and decay after 50 s. It is obviously observed from maps that the better memory ability can be obtained under the stimulation of 532 nm laser with stronger pump fluences.

The power law presented by Wickelgren in 1974 is considered as a suitable model to describe the biological forgetting law, which can be reduced as

(3) $$I=\lambda \times {\left(1+\beta \times t\right)}^{-\Psi }$$

where *I* is the memory strength, *t* is the decay time, *λ* is the state of long‐term memory at *t* = 0 (degree of learning), *β* is a scale parameter, and *ψ* is the forgetting rate. All three of these forgetting processes are well quantified by the Wickelgren's power‐law model (Figure 4h).^[^ ^39^ ^]^ The detailed fitting parameters are given in Table S4 (Supporting Information). Figure 4i plots the values of *λ* and *ψ* under various wavelength stimulations, where the maximum value of *λ* and the minimum value of *ψ* are both obtained at 532 nm stimulations. Figure 4j plots the values of pulse‐density‐dependent *λ* and *ψ*, where *ψ* decreases dramatically as the pulse‐density increases, but *λ* exhibits completely opposite behaviors. It can be understood that the deeper learning (large *λ*) can be caused by intensive learning, in other words, intensive learning can build up a more stable state which also decreases the forgetting ability (low *ψ*). The detailed synaptic performances of a single device are provided in Supporting Information S7.

**Figure 5:**
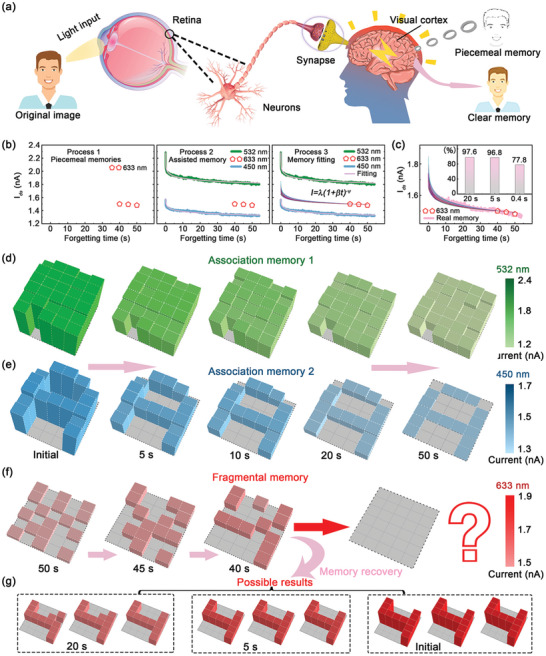
Potential ability of association memory in arrayed optoelectronic synapses. a) The schematics of the overall process of human association memory. b) Association memory of optoelectronic synapse restored with three processes. c) The restored decay currents (stimulated at 633 nm) fitted by Wickelgren's power law. The inset is the statistics of accuracy of memory fitting, which reaches 97.6%, 96.8%, and 77.8% in 20, 5, and 0.4 s, respectively. d) The image of known association memory 1 (stimulated at 532 nm) decays in 50 s. e) The image of known association memory 2 (stimulated at 450 nm) decays in 50 s. f) The schematic of ineffective memory recovery with few known fragmental memory (at 40, 45, 50 s). g) The successful memory recovery (stimulated at 633 nm) with possible results using scientific association memory.

With the understanding of synaptic behaviors of a single device, arrayed MoS_2_ devices can be further explored to imitate the smart memory of human brain. **Figure** **5**a shows the schematics of association memory in human brain evoked by retina, nerve, synapse, and visual cortex. When an image or a scene is recorded by human eyes, the photosensory cells are activated by light stimulus converting optical signals into electrical signals. Then, electrical signals can be transduced via nerves and synapses into the cells of visual cortex resulting in brain behaviors of memorizing, forgetting, and reasoning. When an original image is memorized in brain, the recorded image always becomes blurred as time goes by, and it is hard for a human to remember the clear scene happened in the far past. But utilizing the scientific memory method, the vague recorded image can be easily restored by associating environment characteristics or item specifics at that time, which is called association memory. The associative memory is one of the significant features of artificial intelligence, which helps to connect piecemeal memories in series to make original information restored and reproduced.

Here, we demonstrate the potential ability of association memory in our arrayed optoelectronic synapses based on MoS_2_ monolayers. Figure 5b shows the basic principle of three processes in realizing association memory. In process 1, there are three characteristic photocurrent intensities (at 40, 45, and 50 s) noted as the piecemeal memories, which are stimulated by 633 nm excitation. In process 2, EPSC curves stimulated by 450 and 532 nm excitations are plotted as the well‐known association images, and they are fitted by Wickelgren's power law to obtain the values of *λ* and *ψ*. Because the characteristic piecemeal memories and the decay trend are known, a few possible EPSC curves under 633 nm stimulation can be fitted following the rules in Figure 4i. If the fitted EPSC curves are known, the missing original information can be reappeared, which is the basic concept of our device in realizing the reconstruction of memorized information by association. Figure 5c shows that experiment results and possible fitting curves consist well with each other. Inset shows the statistics of fitting accuracy of photocurrent at 20, 5, and 0.4 s. The fitting accuracies are nearly 100% for restored images at 20 s (97.6%) and 5 s (96.8%), while it is 77.8% for 0.4 s. The detailed fitting results are provided in Note S1 (Supporting Information).

Figure 5d,e shows two examples of association images stimulated by 532 and 450 nm irradiations, respectively, noted as association image 1 and association image 2. The two original images are both stimulated after 8 successive optical stimulus (0.12 mW cm^−2^, 50 ms), and their intensities decrease as time goes by. Association image 1 is a random pattern, where almost all regions are stimulated, and only one unit is left without irradiation. It can be considered as a kind of background information to recover the memory briefly. While association image 2 is a characteristic pattern showing the letter of “A,” which represents the detailed information to inspire the memory.

Figure 5f shows examples of vague memory images with random patterns at the decay time of 40, 45, and 50 s. It is hard to know their original pattern without any inspiration. Utilizing our proposed associative memory method, some possible original patterns stimulated under 633 nm irradiation can be restored at various period times of decay process (20, 5 s and initial) showing the number of “4,” as shown in Figure 5g. If a human wants to recall memories of those far‐off days, it is hard to think out in a direct way. However, if he or she tries to restore the memories by thinking back of a scene or a building at that moment, memory recall will be easier. Therefore, when connected with relevant detailed information, like specific logos, similar sound or characteristic symbols, the deep and vague memory can be easily evoked. Analogy with human association memory, the missing patterns stimulated by 633 nm can be successfully restored in our synaptic devices, with the help of background patterns stimulated by 532 nm and characteristic patterns stimulated by 450 nm.

## 3 Conclusion

In summary, we demonstrate the successful CVD growth of MoS_2_ monolayer array assisted by nucleation islands of gold nanorods. Arrayed MoS_2_ optoelectronic devices own high and low resistance states in photocurrent loops, which are explored as synaptic devices exhibiting high performances in the measurement of EPSC, PPF, STP, LTP. The synaptic abilities of learning and forgetting degrees are systematically investigated by changing the stimulation wavelength and the light power. Interestingly, a 5×6 pixels sensing and processing system is designed to imitate the learning, forgetting, and association memorizing of human brain. Benefiting from the brief information stimulated by 532 nm and detailed information stimulated by 450 nm, the deep and vague memorized patterns recorded by 633 nm stimulation are successfully restored with the restore accuracy reaching 97.6%, 96.8%, and 77.8% at 20, 5, and 0.4 s. Our work achieves a valuable breakthrough in emulating the brain‐like artificial intelligence using arrayed 2D semiconductors, which makes a big step in designing smart retina and exploring complex brain‐like system.

## 4 Experimental Section

### Preparation of Arrayed MoS_2_ Monolayers

Nanohole arrays in photoresist layer on SiO_2_/Si substrate were fabricated by direct laser‐writing photolithography (ARMS SYSTEM UTA‐318), and then 50 nm golden layer was deposited on the substrate to obtain the arrayed gold nanorods. Substrate with arrayed nanorods was placed at the center of a furnance in a cube in vacuum environment. By precisely controlling the weight ratio of powder sources and the velocity of carrier gas, MoS_2_ monolayers were successfully grown when the temperature was increased to 810 °C for 30 min and kept for 5 min. The air flow rate was 70 sccm at the beginning, but reduced at 3–4 sccm after heating.

### Optical and Spectral Measurements

The optical and spectral properties were measured by a home‐built µ‐PL system. An iHR550 Raman spectrometer from Horiba was utilized to measure the PL and Raman spectra with 300 and 1200 g mm^−1^ gratings, respectively. A 532 nm solid‐state laser was also induced to excite samples to obtain the steady‐state spectra. The objective lens is 50× magnifications, and the diameter of the laser spot is ≈1 µm.

### Device Fabrication and Measurements

Typical E‐beam lithography (EBL, Raith 150) and thermal evaporation were used to fabricate a pair of Au/Cr (50 nm/10 nm) electrodes on arrayed MoS_2_ monolayers. Lake Shore Probe Station and Agilent B1500A semiconductor analyzer were used to measure the electrical and optoelectronic properties of the devices at room temperature in vacuum. Pulsed light sources of 450, 532, and 633 nm were chosen to stimulate the synaptic behaviors of devices.

## Conflict of Interest

The authors declare no conflict of interest.

## Supporting information

Supporting Information

## Data Availability

The data that support the findings of this study are available from the corresponding author upon reasonable request.
