# Supplementary material for: Multifunctional Optoelectronic Synapses Based on Arrayed MoS2 Monolayers Emulating Human Association Memory
Source: Adv Sci (Weinh). 2023 Apr 14;10(16):2300120. doi: 10.1002/advs.202300120 (PMC10238179; doi:10.1002/advs.202300120)
Supplement: Supplementary file 1 — Supporting Information [file ADVS-10-2300120-s001.pdf]

ADVANCED SCIENCE
Open Access

Supporting Information

for *Adv. Sci.*, DOI 10.1002/advs.202300120

Multifunctional Optoelectronic Synapses Based on Arrayed MoS_2 Monolayers Emulating Human Association Memory

Ming Huang, Wajid Ali, Liuli Yang, Jianhua Huang, Chengdong Yao, Yunfei Xie, Ronghuan Sun, Chenguang Zhu, Yike Tan, Xiao Liu, Shengman Li, Ziwei Li and Anlian Pan**

Supplementary Material

Multifunctional Optoelectronic Synapses Based on Arrayed MoS_2 Monolayers Emulating Human Association Memory

Ming Huang, Wajid Ali, Liuli Yang, Jianhua Huang, Chengdong Yao, Yunfei Xie, Ronghuan Sun, Chenguang Zhu, Yike Tan, Xiao Liu, Shengman Li, Ziwei Li, Anlian Pan**

Key Laboratory for Micro-Nano Physics and Technology of Hunan Province, Hunan Institute of Optoelectronic Integration, College of Materials Science and Engineering, Hunan University, Changsha, Hunan 410082, P. R. China.

S1. Schematics of CVD growth of arrayed MoS_2.

S2. Optical and spectral characterizations of MoS_2 arrays.

S3. Electrical measurements of devices.

S4. The minimum power consumption measurement.

S5. Simulations of electromagnetic fields at various wavelengths.

S6. EPSC triggered at various excitation densities and wavelengths.

S7. Synaptic performances of a single device.

S8. Depression process of optoelectronic device.

Table S1. Power consumption statistics of 30 different devices.

Table S2. Comparison of Minimum Optical Power Density and Energy Consumption among Synaptic Transistors.

Table S3. Current size and specific value of forgetting current under different power stimulations at three wavelengths.

Table S4. The fitting parameters in the Wickelgren's forgetting model.

Supplementary Note 1

S1. Schematics of CVD growth of arrayed MoS_2.

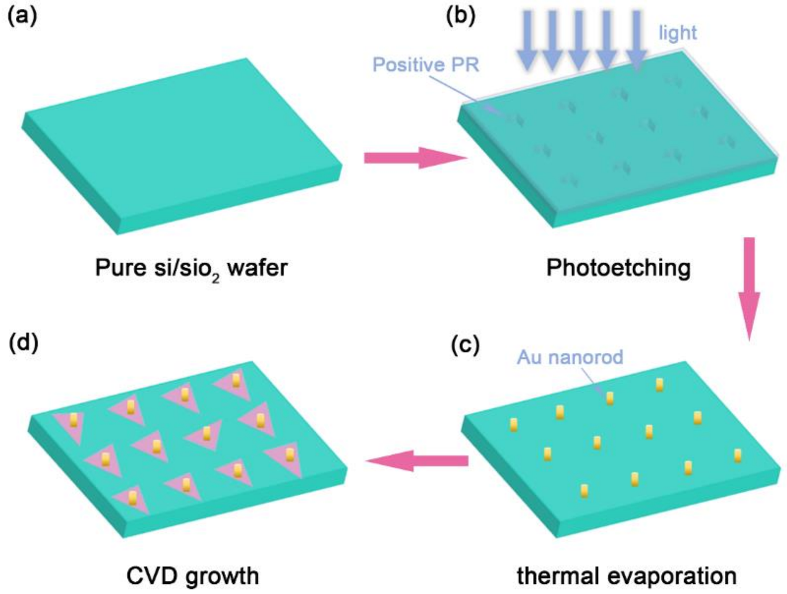

Figure S1. (a) A Si/SiO_2 substrate was prepared after a standard cleaning process. (b) The photoresist was spin-coated on the Si/SiO_2 substrate. Array nanoholes were fabricated by laser direct-writing method. (c) Arrayed gold nanorods were realized after depositing Au film thermal evaporation. (d) Monolayered MoS_2 arrays were successfully grown by chemical vapor deposition with the guide of gold nanorods.

S2. Optical and spectral characterizations of MoS_2 arrays.

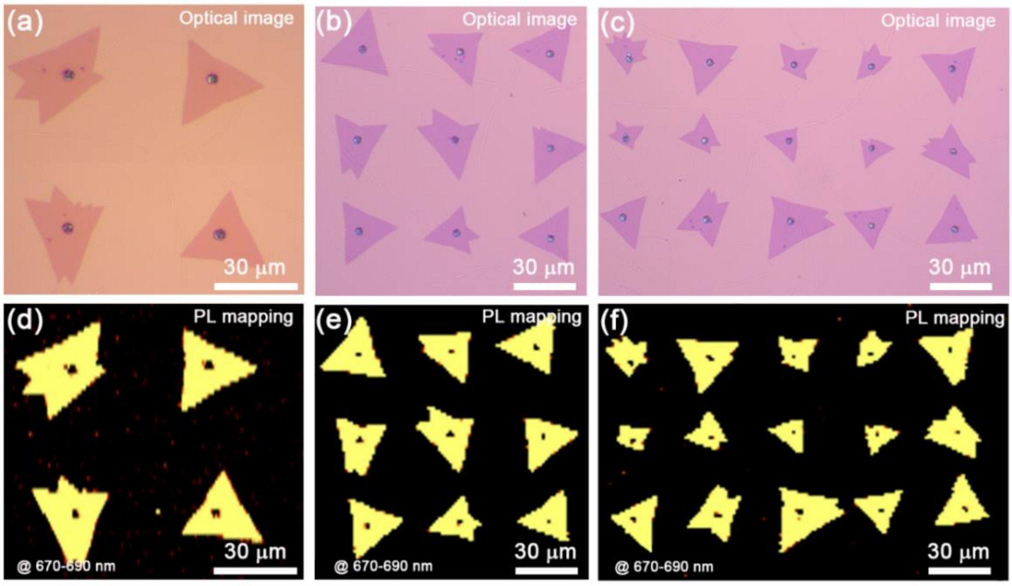

Figure S2. Optical images of 2×2 array (a), 3×3 array (b) and 3×5 array (c) of MoS_2 monolayers. Corresponding PL mapping images 2×2 array (d), 3×3 array (e) and 3×5

array (f). The intensity of MoS_2 flakes are similar confirming the uniform growth of high-quality MoS_2. All scale bars are 30 μm.

S3. Electrical measurements of device.

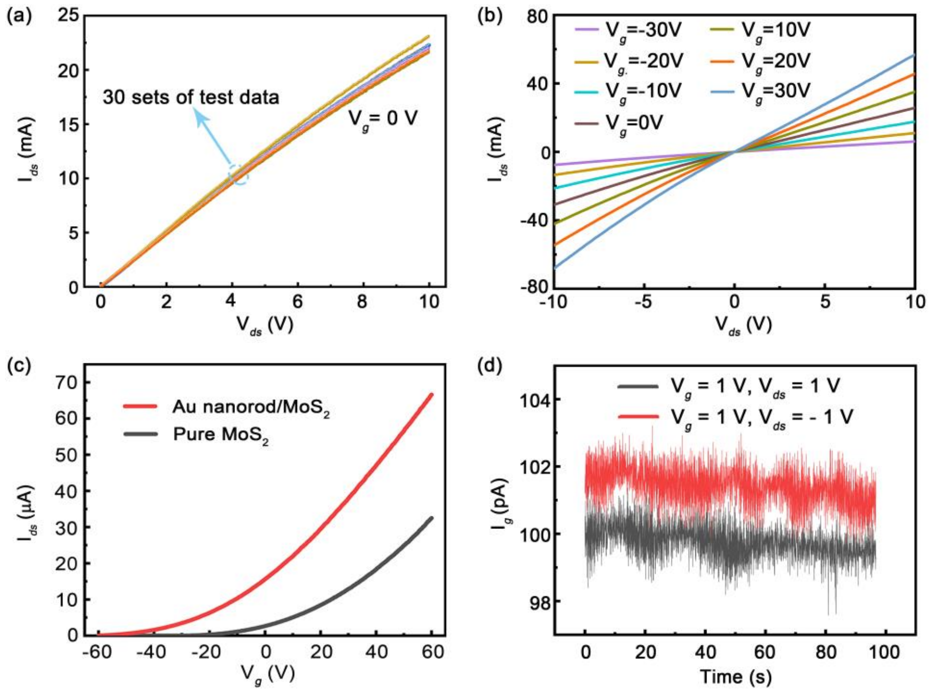

Figure S3. (a) Output characteristic curves of thirty devices working at Vg =0 V in dark environment, where optoelectronic performances of devices are uniform. (b) I_{ds}-V_d curves at different gate voltages ranging from -30 to 30 V in steps of 10 V. All curves are linear indicating the ohmic contact between electrode and MoS_2. (c) The comparision of transfer characteristic curves of pure MoS_2 transistor and as-fabricated Au-nanorod coupled MoS_2 transistor. The threshold voltage of pure MoS_2 transistor is about −10 V, while it shifts to −45 V for as-fabricated Au-nanorod coupled MoS_2 transistor, indicating our sample is highly n-doped MoS_2. The reason of highly n-doped MoS_2 samples may be related to the less Au element doping in MoS_2 flakes during the high-temperature CVD growth. Our experiments consist well with previous work, that gold-nanoparticle guided MoS_2 growth could make highly n-type semiconductor, shifting the threshold of transistor device to a large negative threshold voltage.[1, 2] (d) The leakage current of as-fabricated transistor. Leakage currents of device were detected to be stable at 100 pA (V_g=1 V, V_{ds}=1 V) and 102 pA (V_g=1 V,

V_{ds}=−1 V), which indicates that the leakage current is quite small without affecting the optoelectronic measurement of device.

S4. The minimum power consumption measurement.

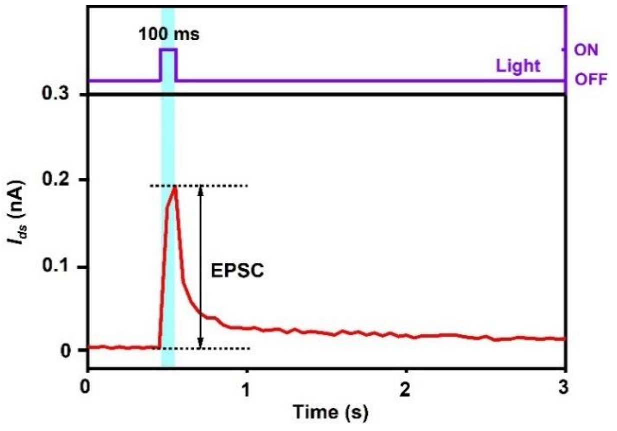

Figure S4. The minimum power consumption measurement under 1.5 μW cm^{-2} with a 100 ms irradiation is 26.9 pJ for a single light stimulus (V_d = 1V, V_g = −60 V).

S5. Simulations of electromagnetic fields at various wavelengths.

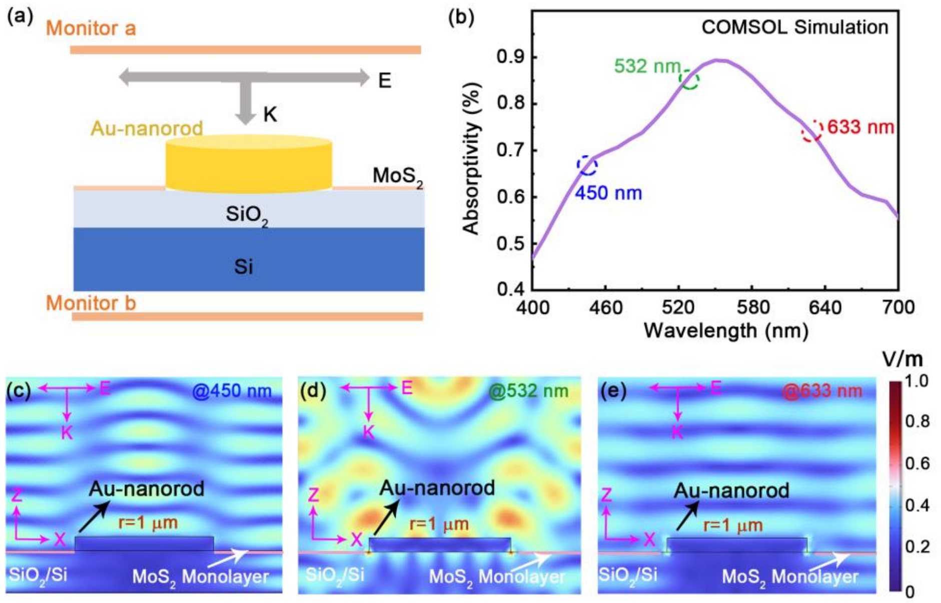

Figure S5. (a) The schematic view of simulated nanostructures in electromagnetic field software. The direction of wave source faces down, and "monitor a" as well as "monitor b" are used to obtain the absorptivity. (b) The normalized absorptivity of Au-nanorod coupled MoS_2 fakes on SiO_2/Si substrate. The main absorption peak is located at around 560 nm, and the excitation wavelength of 532 nm is closer to the

main peak. Side view of electromagnetic field distribution of Au-nanorod coupled MoS_2 at different resonant wavelengths among 450 nm (c), 532 nm (b), and 633 nm (c). The stronger intensity distribution is observed at 532 nm, where the enhanced electromagnetic field is localized around the Au-nanorod, which could promote the light-matter interaction and induce plasmonic “hot”-electron-doping for the improvement of optoelectronic performances. The electromagnetic field experiment is simulated under the COMSOL 6.0 software.

S6. EPSC triggered at various excitation densities and wavelengths.

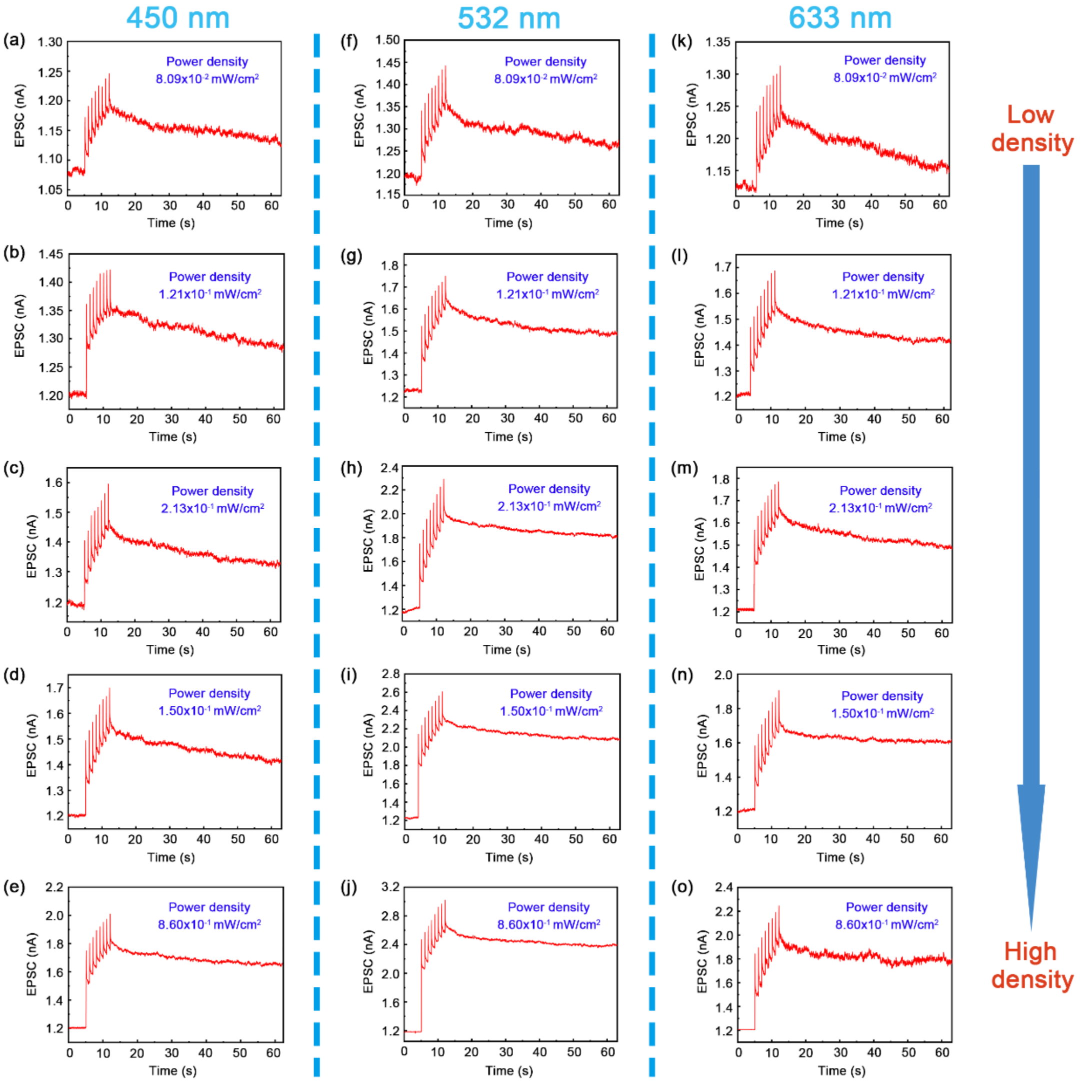

Figure S6. (a-e) EPSC and forgetting curves induced by 8 spikes at different excitation densities. The wavelength is 450 nm. The EPSC increases from 1.25 to 2.11 nA as the spike excitation density increases from 8.09×10^{-2} to 8.60 mW cm^{-2}. (f-j)

EPSC and forgetting curves induced by 8 spikes at different spike excitation densities. The wavelength is 532 nm. The EPSC increases from 1.38 to 3.0 nA as the spike excitation density increases from 8.09×10^{-2} to 8.60 mW cm^{-2}. (k-o) EPSC and forgetting curves induced by 8 spikes at different spike excitation densities. The wavelength is 633 nm. The EPSC increases from 1.31 to 2.24 nA as the spike excitation density increases from 8.09×10^{-2} to 8.60 mW cm^{-2}.

S7. Synaptic performances of a single device.

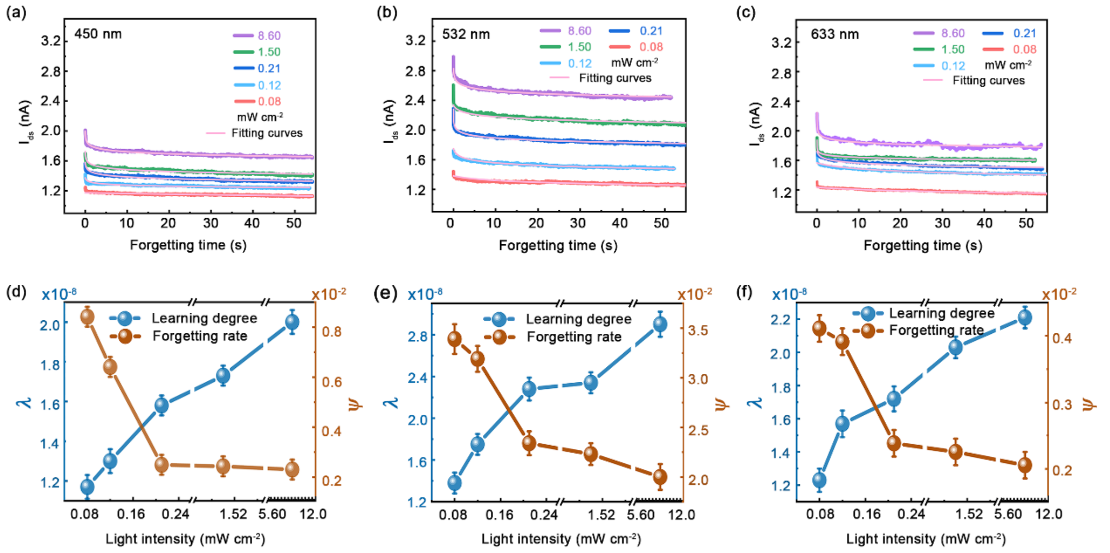

Figure S7. Forgetting curves with various light powers under the stimulation of 450 nm (a), 532 nm (b) and 633 nm (c) irradiations, which are well-fitted by the Wickelgren’s power law. Plots of forgetting rate ψ and learning degree λ at different wavelength of 450 nm (d), 532 nm (e) and 633 nm (f).

S8. Depression process of optoelectronic device.

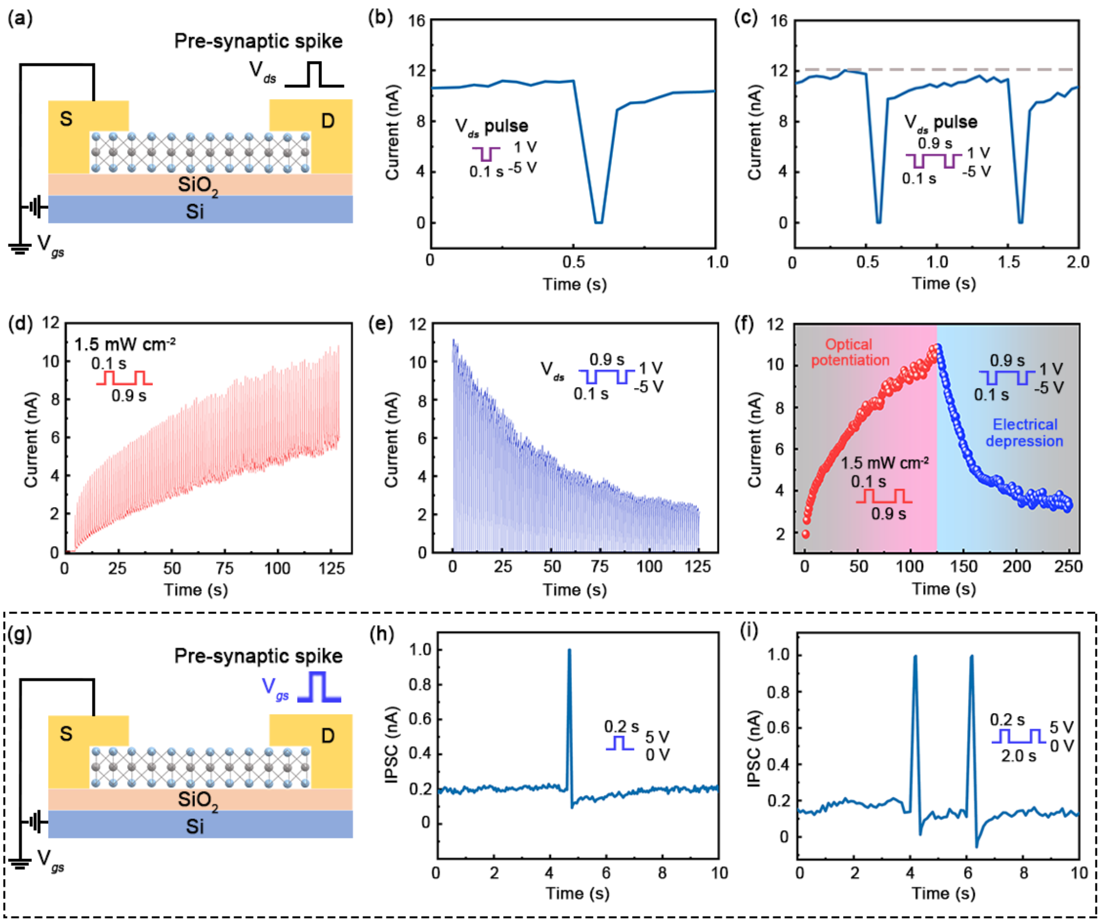

Figure S8. (a) Schematics of optoelectronic synaptic device triggered by pulsed drain voltage. Tests were performed at V_g=0 V, and V_{ds} was input by electrical pulses (pulse width: 0.1 s, pulse interval: 0.9 s). (b) Current of the device triggered by an electrical pulse with the voltage amplitude of − 6 V. Current decreased rapidly under the electrical depression, then tended to recover back after removing the electrical signal. (c) Current of the device triggered by two electrical pulses (voltage amplitude: − 6 V). Dotted line is the current intensity of 12 nA. After the depression process of two electrical pulses, the total trend of current intensity decreased a little. (d) EPSC plasticity using optical pulses for 125 times (power: 1.5 mW cm^{-2}). Current increased from 0 to 11 nA during the process of optical potentiation. (e) Current plasticity using continuous electrical pulses for 125 times (amplitude: −6 V). Current decreased from 11 to 3 nA during the process of electrical depression. (f) The values of the current in two processes of continuous optical and electrical stimulations related to the time

process. (g) Schematics of optoelectronic synaptic device triggered by pulsed gate voltage. All tests were input by electrical gate pulses (V_g=+5 V, pulse width: 0.2 s, pulse interval: 2 s). (h) Current of the device triggered by an electrical pulse. Current was increased rapidly when the gate voltage was switched, then decreased gradually under the electrical depression. After turning-off the voltage, current increased little. (i) Current of the device triggered by two electrical pulses. After repeated electrical gate pulses, the total trend of current intensity decreased gradually.

Table S1. Power consumption statistics of 30 different devices.

	**1**	**2**	**3**	**4**	**5**	**6**
**1**	66.5 pJ	74.0 pJ	77.4 pJ	72.5 pJ	67.5 pJ	62.5 pJ
**2**	91.5 pJ	68.0 pJ	67.0 pJ	87.0 pJ	85.5 pJ	77.0 pJ
**3**	85.5 pJ	67.5 pJ	76.5 pJ	71.5 pJ	76.5 pJ	92.0 pJ
**4**	65.5 pJ	62.0 pJ	62.5 pJ	78.5 pJ	95.5 pJ	85.0 pJ
**5**	92.5 pJ	82.0 pJ	84.5 pJ	79.5 pJ	90.0 pJ	84.0 pJ

We tested the power consumption of 30 devices and obtained an average power consumption of 77.6 pJ for a single stimulus (532 nm, 0.21 mW cm^{-2}).

Table S2. Comparison of Minimum Optical Power Density and Energy Consumption among Synaptic Transistors.

**Materials**	**Optical stimuli wavelength**	**Power consumption**	**Ref.**
Black Phosphorus	280, 660 nm	924 pJ	[3]
MoS_2	455 nm	13 nJ	[4]
MoS_2/ionic liquid	445 nm	13.4 mJ	
TiN_xO_{2-x}/MoS_2	365 nm	450 nJ	[5]
$Ag/MoSe_2/Bi_2Se/ITO$	790 nm	1 nJ	[6]
SWNT/Gr	405 nm	250 nJ	[7]
single-walled CNT/graphene	405 nm	150 nJ	
pentacene/PMMA/$CsPbBr_3$	365 nm	1.4 nJ	[8]
C8-BTBT/PAN	360 nm	420 pJ	[9]
$CsPbBr_3$ QDs+PQT-12	100 nm	650 pJ	[10]
$IGZO/SiO_x/SiN_x$	375 nm	123 pJ	[11]
$(PEA)_2SnI_4$	470 nm	240 nJ	[12]

IGZO	380-385 nm	100 nJ	[13]
ITO/chitosan	400 nm	3.92 mJ	[14]
**MoS_2 Array**	**450, 532, 633 nm**	**26.9 pJ**	**this work**

Table S3. Current size and specific value of forgetting current under different power stimulations at three wavelengths.

**Wavelength**	**Power density (mW/cm^2)**	**Before Stimulate**	**Initial**	**After 5 s**	**After 10 s**	**After 20 s**	**After 50 s**
450 nm	8.9×10^{-2}	1.07 nA	1.25	1.17	1.16	1.15	1.13
	1.21×10^{-1}	1.20 nA	1.42	1.34	1.33	1.32	1.28
	2.13×10^{-1}	1.18 nA	1.59	1.41	1.39	1.37	1.32
	1.50	1.22 nA	1.70	1.51	1.49	1.47	1.41
	8.60	1.20 nA	2.02	1.75	1.72	1.69	1.65
532 nm	8.9×10^{-2}	1.14 nA	1.44	1.31	1.30	1.28	1.26
	1.21×10^{-1}	1.22 nA	1.73	1.58	1.55	1.52	1.48
	2.13×10^{-1}	1.18 nA	2.28	1.93	1.89	1.87	1.80
	1.50	1.22 nA	2.60	2.23	2.19	2.15	2.10
	8.60	1.20 nA	3.00	2.56	2.52	2.48	2.43
633 nm	8.9×10^{-2}	1.13 nA	1.31	1.21	1.20	1.19	1.15
	1.21×10^{-1}	1.22 nA	1.68	1.50	1.48	1.45	1.41
	2.13×10^{-1}	1.20 nA	1.79	1.59	1.57	1.54	1.48
	1.50	1.21 nA	1.91	1.65	1.63	1.62	1.60
	8.60	1.20 nA	2.24	1.89	1.84	1.81	1.78

Table S4. The fitting parameters in the Wickelgren's forgetting model.

**Wavelength**	**Power density (mW/cm^2)**	λ	ψ	β
450 nm	8.9×10^{-2}	1.1738×10^{-9}	0.0841	0.01
	1.21×10^{-1}	1.304×10^{-9}	0.06393	0.0261
	2.13×10^{-1}	1.5795×10^{-9}	0.02447	20.235
	1.50	1.7256×10^{-9}	0.02392	55
	8.60	1.998×10^{-9}	0.02256	71.85

532 nm	8.9×10^{-2}	1.383×10^{-9}	0.03393	0.289
	1.21×10^{-1}	1.752×10^{-9}	0.03187	3.87058
	2.13×10^{-1}	2.281×10^{-9}	0.02341	286.9
	1.50	2.337×10^{-9}	0.02233	2.194
	8.60	2.9×10^{-9}	0.01995	96.691
633 nm	8.9×10^{-2}	1.2342×10^{-9}	0.04107	0.07342
	1.21×10^{-1}	1.566×10^{-9}	0.0391	0.27738
	2.13×10^{-1}	1.724×10^{-9}	0.02383	5.79
	1.50	1.931×10^{-9}	0.0225	109
	8.60	2.21×10^{-9}	0.0205	615

Supplementary Note 1

According to the above data, it is clearly observed that when the device is excited under the light stimulation of 633 nm laser, the value of learning degree λ of the forgetting curve is less than the value under 532 nm stimulations, larger than the value under 450 nm stimulations, and the value of forgetting rate ψ is also between them, but the value is larger than that under 450 nm stimulations and smaller than that under 532 nm stimulations.

Figure 5b shows the basic principle of three processes in realizing association memory. In process 1, there are three characteristic photocurrent intensities (at 40 s, 45 s and 50 s) noted as the piecemeal memories, which are stimulated by 633 nm excitation. In process 2, EPSC curves stimulated by 450 nm and 532 nm excitations are plotted as the known association information, and they are fitted by Wickelgren’s power law to obtain the values of λ and ψ. Because the characteristic piecemeal memories and the decay trend are known, a few possible EPSC curves under 633 nm stimulation can be fitted following the rules in Fig. 4i

$$I = \lambda \times (1 + \beta \times t)^{-\psi}$$

First, we fitted the forgetting curves stimulated by 532 nm and 450 nm wavelengths, and obtained the values in the following table respectively

Section 1. Fitting parameters of forgetting curves at different wavelengths.

**Wavelength**	λ	ψ	β
450	1.5795×10^{-9}	0.02447	20.235
633	/	/	/
532	2.281×10^{-9}	0.02341	286.9

Next, according to the constraints, we perform curve fitting on the three known data points of the 633 nm forgetting curve, when λ is fixed at 1.6×10^{-9}, a fitting curve can be obtained, then λ is fixed at 1.7, 1.8, 1.9, 2.0, 2.1 and 2.2×10^{-9} respectively, and seven fitting results can be obtained. Similarly, we also fix the value of the parameter ψ, and a series of fitting curves can be obtained. However, the fitting curve error of only fixing one parameter is too large, so we fixed the two parameters in the following fitting.

Two parameters λ and ψ are fixed simultaneously, and a series of fitting curves can be obtained

1) Fix λ to 1.6×10^{-9} and ψ to 0.0244, the fitting curve is

$$I = 1.6\times10^{-9}\times(1+0.338t)^{-0.0244}$$

2) Fix λ to 1.7×10^{-9} and ψ to 0.0244, the fitting curve is

$$I = 1.7\times10^{-9}\times(1+4.105t)^{-0.0244}$$

3) Fix λ to 1.8×10^{-9} and ψ to 0.0244, the fitting curve is

$$I = 1.8\times10^{-9}\times(1+42.92681t)^{-0.0244}$$

4) Fix λ to 1.9×10^{-9} and ψ to 0.0244, the fitting curve is

$$I = 1.9\times10^{-9}\times(1+393.7896t)^{-0.0244}$$

5) Fix λ to 2.0×10^{-9} and ψ to 0.0244, the fitting curve is

$$I = 2.0\times10^{-9}\times(1+3222.94027t)^{-0.0244}$$

According to the above fitting results, we can find that the fourth and fifth fitting curves have distortion, mainly because the β fitting value is too large, resulting in the meaningless λ and ψ, so the fourth and fifth fitting results are removed. At the same

time, in order to ensure the fitting data close to the real memory value as much as possible, so the first fitting result is also removed. We divide λ from 1.65 to 1.85 into 21 pieces with 0.01 intervals, ψ from 0.0244 to 0.0234 into 6, with 0.002 intervals We get 126 fitting curves in the following table in this way.

Section 2. 126 different fitting results (ψ into 10, with 0.001 intervals, λ into 6, with 0.01 $\times 10^{-9}$ intervals).

**Number**	λ	ψ	β	**curve**
**1**	1.65×10^{-9}	0.0244	1.19235	$I = 1.65\times10^{-9}\times(1+1.192t)^{-0.0244}$
**2**	1.66×10^{-9}	0.0244	1.53349	$I = 1.66\times10^{-9}\times(1+1.533t)^{-0.0244}$
**3**	1.67×10^{-9}	0.0244	1.1967	$I = 1.67\times10^{-9}\times(1+1.197t)^{-0.0244}$
**4**	1.68×10^{-9}	0.0244	2.52	$I = 1.68\times10^{-9}\times(1+2.52t)^{-0.0244}$
**5**	1.69×10^{-9}	0.0244	3.219	$I = 1.69\times10^{-9}\times(1+3.219t)^{-0.0244}$
**6**	1.70×10^{-9}	0.0244	4.105	$I = 1.70\times10^{-9}\times(1+4.105t)^{-0.0244}$
**7**	1.71×10^{-9}	0.0244	5.226	$I = 1.71\times10^{-9}\times(1+5.226t)^{-0.0244}$
**8**	1.72×10^{-9}	0.0244	6.64	$I = 1.72\times10^{-9}\times(1+6.64t)^{-0.0244}$
**9**	1.73×10^{-9}	0.0244	8.43	$I = 1.73\times10^{-9}\times(1+8.43t)^{-0.0244}$
**10**	1.74×10^{-9}	0.0244	10.68	$I = 1.74\times10^{-9}\times(1+10.68t)^{-0.0244}$
**11**	1.75×10^{-9}	0.0244	13.52	$I = 1.75\times10^{-9}\times(1+13.52t)^{-0.0244}$
**12**	1.76×10^{-9}	0.0244	17.07	$I = 1.76\times10^{-9}\times(1+17.07t)^{-0.0244}$
**13**	1.77×10^{-9}	0.0244	21.55	$I = 1.77\times10^{-9}\times(1+21.55t)^{-0.0244}$
**14**	1.78×10^{-9}	0.0244	27.15	$I = 1.78\times10^{-9}\times(1+27.15t)^{-0.0244}$
**15**	1.79×10^{-9}	0.0244	34.16	$I = 1.79\times10^{-9}\times(1+34.16t)^{-0.0244}$
**16**	1.80×10^{-9}	0.0244	42.93	$I = 1.80\times10^{-9}\times(1+42.93t)^{-0.0244}$
**17**	1.81×10^{-9}	0.0244	53.87	$I = 1.81\times10^{-9}\times(1+53.87t)^{-0.0244}$
**18**	1.82×10^{-9}	0.0244	67.528	$I = 1.82\times10^{-9}\times(1+67.528t)^{-0.0244}$
**19**	1.83×10^{-9}	0.0244	84.53	$I = 1.83\times10^{-9}\times(1+84.53t)^{-0.0244}$
**20**	1.84×10^{-9}	0.0244	105.69	$I = 1.84\times10^{-9}\times(1+105.69t)^{-0.0244}$
**21**	1.85×10^{-9}	0.0244	131.82	$I = 1.85\times10^{-9}\times(1+131.82t)^{-0.0244}$
**22**	1.65×10^{-9}	0.0242	1.233	$I = 1.65\times10^{-9}\times(1+1.233t)^{-\ 0.0242}$
**23**	1.66×10^{-9}	0.0242	1.589	$I = 1.66\times10^{-9}\times(1+1.589t)^{-0.0242}$
**24**	1.67×10^{-9}	0.0242	2.0427	$I = 1.67\times10^{-9}\times(1+2.043t)^{-0.0242}$
**25**	1.68×10^{-9}	0.0242	2.62	$I = 1.68\times10^{-9}\times(1+2.62t)^{-0.0242}$
**26**	1.69×10^{-9}	0.0242	3.355	$I = 1.69\times10^{-9}\times(1+3.355t)^{-0.0242}$
**27**	1.70×10^{-9}	0.0242	4.287	$I = 1.70\times10^{-9}\times(1+4.287t)^{-0.0242}$
**28**	1.71×10^{-9}	0.0242	5.4684	$I = 1.71\times10^{-9}\times(1+5.468t)^{-0.0242}$
**29**	1.72×10^{-9}	0.0242	6.964	$I = 1.72\times10^{-9}\times(1+6.964t)^{-0.0242}$
**30**	1.73×10^{-9}	0.0242	8.855	$I = 1.73\times10^{-9}\times(1+8.855t)^{-0.0242}$
**31**	1.74×10^{-9}	0.0242	11.242	$I = 1.74\times10^{-9}\times(1+11.242t)^{-0.0242}$
**32**	1.75×10^{-9}	0.0242	14.252	$I = 1.75\times10^{-9}\times(1+14.252t)^{-0.0242}$

**33**	1.76×10^{-9}	0.0242	18.041	$I = 1.76{\times}10^{-9}{\times}(1+18.041t)^{-0.0242}$
**34**	1.77×10^{-9}	0.0242	22.81	$I = 1.77{\times}10^{-9}{\times}(1+22.81t)^{-0.0242}$
**35**	1.78×10^{-9}	0.0242	28.7905	$I = 1.78{\times}10^{-9}{\times}(1+28.791t)^{-0.0242}$
**36**	1.79×10^{-9}	0.0242	36.30	$I = 1.79{\times}10^{-9}{\times}(1+36.30t)^{-0.0242}$
**37**	1.80×10^{-9}	0.0242	45.69	$I = 1.80{\times}10^{-9}{\times}(1+45.69t)^{-0.0242}$
**38**	1.81×10^{-9}	0.0242	57.458	$I = 1.81{\times}10^{-9}{\times}(1+57.458t)^{-0.0242}$
**39**	1.82×10^{-9}	0.0242	72.15	$I = 1.82{\times}10^{-9}{\times}(1+72.15t)^{-0.0242}$
**40**	1.83×10^{-9}	0.0242	90.5	$I = 1.83{\times}10^{-9}{\times}(1+90.5t)^{-0.0242}$
**41**	1.84×10^{-9}	0.0242	113.3	$I = 1.84{\times}10^{-9}{\times}(1+113.3t)^{-0.0242}$
**42**	1.85×10^{-9}	0.0242	141.8	$I = 1.85{\times}10^{-9}{\times}(1+141.8t)^{-0.0242}$
**43**	1.65×10^{-9}	0.0240	1.2759	$I = 1.65{\times}10^{-9}{\times}(1+1.276t)^{-0.0240}$
**44**	1.66×10^{-9}	0.0240	1.6475	$I = 1.66{\times}10^{-9}{\times}(1+1.648t)^{-0.0240}$
**45**	1.67×10^{-9}	0.0240	2.122	$I = 1.67{\times}10^{-9}{\times}(1+2.122t)^{-0.0240}$
**46**	1.68×10^{-9}	0.0240	2.7276	$I = 1.68{\times}10^{-9}{\times}(1+2.728t)^{-0.0240}$
**47**	1.69×10^{-9}	0.0240	3.4989	$I = 1.69{\times}10^{-9}{\times}(1+3.50t)^{-0.0240}$
**48**	1.70×10^{-9}	0.0240	4.48	$I = 1.70{\times}10^{-9}{\times}(1+4.48t)^{-0.0240}$
**49**	1.71×10^{-9}	0.0240	5.726	$I = 1.71{\times}10^{-9}{\times}(1+5.726t)^{-0.0240}$
**50**	1.72×10^{-9}	0.0240	7.307	$I = 1.72{\times}10^{-9}{\times}(1+7.307t)^{-0.0240}$
**51**	1.73×10^{-9}	0.0240	9.3092	$I = 1.73{\times}10^{-9}{\times}(1+9.309t)^{-0.0240}$
**52**	1.74×10^{-9}	0.0240	11.842	$I = 1.74{\times}10^{-9}{\times}(1+11.842t)^{-0.0240}$
**53**	1.75×10^{-9}	0.0240	15.041	$I = 1.75{\times}10^{-9}{\times}(1+15.041t)^{-0.0240}$
**54**	1.76×10^{-9}	0.0240	18.889	$I = 1.76{\times}10^{-9}{\times}(1+18.889t)^{-0.0240}$
**55**	1.77×10^{-9}	0.0240	24.12	$I = 1.77{\times}10^{-9}{\times}(1+24.12t)^{-0.0240}$
**56**	1.78×10^{-9}	0.0240	30.126	$I = 1.78{\times}10^{-9}{\times}(1+30.126t)^{-0.0240}$
**57**	1.79×10^{-9}	0.0240	38.25	$I = 1.79{\times}10^{-9}{\times}(1+38.25t)^{-0.0240}$
**58**	1.80×10^{-9}	0.0240	50.298	$I = 1.80{\times}10^{-9}{\times}(1+50.298t)^{-0.0240}$
**59**	1.81×10^{-9}	0.0240	61.956	$I = 1.81{\times}10^{-9}{\times}(1+61.956t)^{-0.0240}$
**60**	1.82×10^{-9}	0.0240	82.254	$I = 1.82{\times}10^{-9}{\times}(1+82.254t)^{-0.0240}$
**61**	1.83×10^{-9}	0.0240	101.2	$I = 1.83{\times}10^{-9}{\times}(1+101.2t)^{-0.0240}$
**62**	1.84×10^{-9}	0.0240	119.5	$I = 1.84{\times}10^{-9}{\times}(1+119.5t)^{-0.0240}$
**63**	1.85×10^{-9}	0.0240	145.8	$I = 1.85{\times}10^{-9}{\times}(1+145.8t)^{-0.0240}$
**64**	1.65×10^{-9}	0.0238	1.3325	$I = 1.65{\times}10^{-9}{\times}(1+1.333t)^{-0.0238}$
**65**	1.66×10^{-9}	0.0238	1.825	$I = 1.66{\times}10^{-9}{\times}(1+1.825t)^{-0.0238}$
**66**	1.67×10^{-9}	0.0238	2.211	$I = 1.67{\times}10^{-9}{\times}(1+2.211t)^{-0.0238}$
**67**	1.68×10^{-9}	0.0238	3.012	$I = 1.68{\times}10^{-9}{\times}(1+3.012t)^{-0.0238}$
**68**	1.69×10^{-9}	0.0238	3.854	$I = 1.69{\times}10^{-9}{\times}(1+3.854t)^{-0.0238}$
**69**	1.70×10^{-9}	0.0238	5.015	$I = 1.70{\times}10^{-9}{\times}(1+5.015t)^{-0.0238}$
**70**	1.71×10^{-9}	0.0238	6.55	$I = 1.71{\times}10^{-9}{\times}(1+6.55t)^{-0.0238}$
**71**	1.72×10^{-9}	0.0238	8.256	$I = 1.72{\times}10^{-9}{\times}(1+8.256t)^{-0.0238}$
**72**	1.73×10^{-9}	0.0238	10.2	$I = 1.73{\times}10^{-9}{\times}(1+10.2t)^{-0.0238}$
**73**	1.74×10^{-9}	0.0238	14.2	$I = 1.74{\times}10^{-9}{\times}(1+14.2t)^{-0.0238}$
**74**	1.75×10^{-9}	0.0238	15.98	$I = 1.75{\times}10^{-9}{\times}(1+15.98t)^{-0.0238}$
**75**	1.76×10^{-9}	0.0238	20.12	$I = 1.76{\times}10^{-9}{\times}(1+20.12t)^{-0.0238}$

**76**	1.77×10^{-9}	0.0238	26.589	$I = 1.77\times10^{-9}\times(1+26.589t)^{-0.0238}$
**77**	1.78×10^{-9}	0.0238	33.24	$I = 1.78\times10^{-9}\times(1+33.24t)^{-0.0238}$
**78**	1.79×10^{-9}	0.0238	41.39	$I = 1.79\times10^{-9}\times(1+41.39t)^{-0.0238}$
**79**	1.80×10^{-9}	0.0238	55.87	$I = 1.80\times10^{-9}\times(1+55.87t)^{-0.0238}$
**80**	1.81×10^{-9}	0.0238	70.684	$I = 1.81\times10^{-9}\times(1+70.684t)^{-0.0238}$
**81**	1.82×10^{-9}	0.0238	89.955	$I = 1.82\times10^{-9}\times(1+89.955t)^{-0.0238}$
**82**	1.83×10^{-9}	0.0238	105.68	$I = 1.83\times10^{-9}\times(1+105.68t)^{-0.0238}$
**83**	1.84×10^{-9}	0.0238	121.6	$I = 1.84\times10^{-9}\times(1+121.6t)^{-0.0238}$
**84**	1.85×10^{-9}	0.0238	151.6	$I = 1.85\times10^{-9}\times(1+151.6t)^{-0.0238}$
**85**	1.65×10^{-9}	0.0236	1.458	$I = 1.65\times10^{-9}\times(1+1.458t)^{-0.0236}$
**86**	1.66×10^{-9}	0.0236	1.998	$I = 1.66\times10^{-9}\times(1+1.998t)^{-0.0236}$
**87**	1.67×10^{-9}	0.0236	2.354	$I = 1.67\times10^{-9}\times(1+2.354t)^{-0.0236}$
**88**	1.68×10^{-9}	0.0236	4.012	$I = 1.68\times10^{-9}\times(1+4.012t)^{-0.0236}$
**89**	1.69×10^{-9}	0.0236	4.25	$I = 1.69\times10^{-9}\times(1+4.25t)^{-0.0236}$
**90**	1.70×10^{-9}	0.0236	5.96	$I = 1.70\times10^{-9}\times(1+5.96t)^{-0.0236}$
**91**	1.71×10^{-9}	0.0236	6.85	$I = 1.71\times10^{-9}\times(1+6.85t)^{-0.0236}$
**92**	1.72×10^{-9}	0.0236	8.95	$I = 1.72\times10^{-9}\times(1+8.95t)^{-0.0236}$
**93**	1.73×10^{-9}	0.0236	11.25	$I = 1.73\times10^{-9}\times(1+11.25t)^{-0.0236}$
**94**	1.74×10^{-9}	0.0236	15.768	$I = 1.74\times10^{-9}\times(1+15.768t)^{-0.0236}$
**95**	1.75×10^{-9}	0.0236	18.55	$I = 1.75\times10^{-9}\times(1+18.55t)^{-0.0236}$
**96**	1.76×10^{-9}	0.0236	22.696	$I = 1.76\times10^{-9}\times(1+22.696t)^{-0.0236}$
**97**	1.77×10^{-9}	0.0236	29.36	$I = 1.77\times10^{-9}\times(1+29.36t)^{-0.0236}$
**98**	1.78×10^{-9}	0.0236	38.90	$I = 1.78\times10^{-9}\times(1+38.90t)^{-0.0236}$
**99**	1.79×10^{-9}	0.0236	50.41	$I = 1.79\times10^{-9}\times(1+50.41t)^{-0.0236}$
**100**	1.80×10^{-9}	0.0236	66.10	$I = 1.80\times10^{-9}\times(1+66.10t)^{-0.0236}$
**101**	1.81×10^{-9}	0.0236	74.85	$I = 1.81\times10^{-9}\times(1+74.85t)^{-0.0236}$
**102**	1.82×10^{-9}	0.0236	90.0	$I = 1.82\times10^{-9}\times(1+90.0t)^{-0.0236}$
**103**	1.83×10^{-9}	0.0236	110.1	$I = 1.83\times10^{-9}\times(1+110.1t)^{-0.0236}$
**104**	1.84×10^{-9}	0.0236	129.3	$I = 1.84\times10^{-9}\times(1+129.3t)^{-0.0236}$
**105**	1.85×10^{-9}	0.0236	153.1	$I = 1.85\times10^{-9}\times(1+153.1t)^{-0.0236}$
**106**	1.65×10^{-9}	0.0234	1.68	$I = 1.65\times10^{-9}\times(1+1.68t)^{-0.0234}$
**107**	1.66×10^{-9}	0.0234	2.223	$I = 1.66\times10^{-9}\times(1+2.223t)^{-0.0234}$
**108**	1.67×10^{-9}	0.0234	3.214	$I = 1.67\times10^{-9}\times(1+3.214t)^{-0.0234}$
**109**	1.68×10^{-9}	0.0234	4.98	$I = 1.68\times10^{-9}\times(1+4.98t)^{-0.0234}$
**110**	1.69×10^{-9}	0.0234	5.14	$I = 1.69\times10^{-9}\times(1+5.14t)^{-0.0234}$
**111**	1.70×10^{-9}	0.0234	6.25	$I = 1.70\times10^{-9}\times(1+6.25t)^{-0.0234}$
**112**	1.71×10^{-9}	0.0234	7.01	$I = 1.71\times10^{-9}\times(1+7.01t)^{-0.0234}$
**113**	1.72×10^{-9}	0.0234	9.22	$I = 1.72\times10^{-9}\times(1+9.22t)^{-0.0234}$
**114**	1.73×10^{-9}	0.0234	13.21	$I = 1.73\times10^{-9}\times(1+13.21t)^{-0.0234}$
**115**	1.74×10^{-9}	0.0234	16.85	$I = 1.74\times10^{-9}\times(1+16.85t)^{-0.0234}$
**116**	1.75×10^{-9}	0.0234	20.14	$I = 1.75\times10^{-9}\times(1+20.14t)^{-0.0234}$
**117**	1.76×10^{-9}	0.0234	24.58	$I = 1.76\times10^{-9}\times(1+24.58t)^{-0.0234}$
**118**	1.77×10^{-9}	0.0234	31.25	$I = 1.77\times10^{-9}\times(1+31.25t)^{-0.0234}$

**119**	1.78×10^{-9}	0.0234	45.88	$I = 1.78\times10^{-9}\times(1+45.88t)^{-0.0234}$
**120**	1.79×10^{-9}	0.0234	55.49	$I = 1.79\times10^{-9}\times(1+55.49t)^{-0.0234}$
**121**	1.80×10^{-9}	0.0234	70.82	$I = 1.80\times10^{-9}\times(1+70.82t)^{-0.0234}$
**122**	1.81×10^{-9}	0.0234	80.64	$I = 1.81\times10^{-9}\times(1+80.64t)^{-0.0234}$
**123**	1.82×10^{-9}	0.0234	99.92	$I = 1.82\times10^{-9}\times(1+99.92t)^{-0.0234}$
**124**	1.83×10^{-9}	0.0234	115.2896	$I = 1.83\times10^{-9}\times(1+115.290t)^{-0.0234}$
**125**	1.84×10^{-9}	0.0234	135.7441	$I = 1.84\times10^{-9}\times(1+135.744t)^{-0.0234}$
**126**	1.85×10^{-9}	0.0234	169.78	$I = 1.85\times10^{-9}\times(1+169.78t)^{-0.0234}$

According to the results in the above table, we can get the current (Memory contents) around the time when forgetting begins to occur.

Section 3. Fitting memory versus real memory

**Number**	Real value **(1.54 nA)**	Real value **(1.59 nA)**	Real value **(1.646 nA)**
	**Fitting value (20 s)**	**Fitting value (5 s)**	**Initial（0.426s）**
**1**	1.526	1.574	1.634
**2**	1.526	1.575	1.640
**3**	1.544	1.593	1.653
**4**	1.526	1.576	1.650
**5**	1.526	1.577	1.655
**6**	1.526	1.577	1.659
**7**	1.526	1.578	1.662
**8**	1.526	1.578	1.665
**9**	1.526	1.578	1.667
**10**	1.526	1.578	1.669
**11**	1.526	1.578	1.670
**12**	1.526	1.579	1.672
**13**	1.526	1.579	1.673
**14**	1.526	1.579	1.673
**15**	1.526	1.579	1.674
**16**	1.526	1.579	1.675
**17**	1.526	1.579	1.675
**18**	1.526	1.579	1.675
**19**	1.526	1.579	1.676
**20**	1.526	1.579	1.676
**21**	1.526	1.579	1.676
**22**	1.525	1.573	1.633
**23**	1.526	1.574	1.639
**24**	1.526	1.575	1.645
**25**	1.526	1.576	1.650
**26**	1.526	1.576	1.654

**27**	1.526	1.577	1.658
**28**	1.526	1.577	1.661
**29**	1.526	1.577	1.664
**30**	1.526	1.578	1.666
**31**	1.526	1.578	1.668
**32**	1.526	1.578	1.669
**33**	1.526	1.578	1.670
**34**	1.526	1.578	1.671
**35**	1.526	1.578	1.672
**36**	1.526	1.578	1.673
**37**	1.526	1.578	1.673
**38**	1.526	1.578	1.674
**39**	1.526	1.578	1.674
**40**	1.526	1.578	1.674
**41**	1.526	1.578	1.674
**42**	1.526	1.578	1.675
**43**	1.525	1.573	1.633
**44**	1.525	1.574	1.639
**45**	1.525	1.575	1.644
**46**	1.526	1.575	1.649
**47**	1.526	1.576	1.653
**48**	1.526	1.576	1.657
**49**	1.526	1.576	1.660
**50**	1.526	1.577	1.663
**51**	1.526	1.577	1.665
**52**	1.526	1.577	1.666
**53**	1.526	1.577	1.668
**54**	1.526	1.578	1.669
**55**	1.526	1.577	1.670
**56**	1.526	1.578	1.671
**57**	1.526	1.578	1.672
**58**	1.525	1.576	1.671
**59**	1.526	1.577	1.672
**60**	1.524	1.575	1.670
**61**	1.524	1.576	1.671
**62**	1.527	1.578	1.674
**63**	1.528	1.579	1.675
**64**	1.525	1.572	1.632
**65**	1.523	1.571	1.637
**66**	1.525	1.574	1.644
**67**	1.523	1.573	1.647
**68**	1.524	1.573	1.651
**69**	1.523	1.573	1.654

**70**	1.522	1.573	1.657
**71**	1.523	1.573	1.659
**72**	1.524	1.575	1.662
**73**	1.521	1.572	1.661
**74**	1.525	1.576	1.666
**75**	1.526	1.577	1.668
**76**	1.524	1.575	1.667
**77**	1.525	1.576	1.668
**78**	1.525	1.576	1.670
**79**	1.523	1.574	1.668
**80**	1.523	1.574	1.668
**81**	1.523	1.574	1.668
**82**	1.525	1.576	1.671
**83**	1.528	1.580	1.674
**84**	1.529	1.580	1.675
**85**	1.523	1.570	1.532
**86**	1.521	1.569	1.636
**87**	1.524	1.573	1.643
**88**	1.514	1.563	1.641
**89**	1.521	1.571	1.649
**90**	1.518	1.568	1.650
**91**	1.522	1.572	1.656
**92**	1.522	1.572	1.657
**93**	1.522	1.572	1.660
**94**	1.519	1.569	1.658
**95**	1.522	1.572	1.662
**96**	1.523	1.574	1.664
**97**	1.523	1.573	1.665
**98**	1.521	1.572	1.664
**99**	1.520	1.571	1.663
**100**	1.519	1.570	1.662
**101**	1.523	1.574	1.667
**102**	1.525	1.576	1.669
**103**	1.526	1.577	1.670
**104**	1.529	1.579	1.673
**105**	1.531	1.582	1.676
**106**	1.519	1.566	1.629
**107**	1.518	1.566	1.634
**108**	1.514	1.563	1.637
**109**	1.508	1.557	1.636
**110**	1.516	1.565	1.645
**111**	1.518	1.567	1.649

**112**	1.523	1.572	1.656
**113**	1.522	1.572	1.657
**114**	1.518	1.568	1.655
**115**	1.518	1.568	1.657
**116**	1.521	1.571	1.660
**117**	1.522	1.572	1.662
**118**	1.522	1.572	1.663
**119**	1.517	1.567	1.658
**120**	1.519	1.569	1.661
**121**	1.519	1.569	1.661
**122**	1.523	1.573	1.665
**123**	1.523	1.574	1.666
**124**	1.527	1.577	1.670
**125**	1.529	1.580	1.673
**126**	1.529	1.580	1.673

According to the fitting results, we can calculate the current value in 20s, 5s and the initial value (memory value) in turn. In the model designed by us, we can calculate the maximum error of 0.032 nA, 0.027 nA and 0.0296 nA at 20s, 5s and the beginning, respectively. We consider the error within 0.025 nA to be reasonable, therefore, the accuracy of memory fitting reached 97.62%, 96.83% and 77.78% in 20s, 5s and initial, respectively.

Reference

[1] Z. Luo, Y. Xie, Z. Li, Y. Wang, L. Li, Z. Luo, C. Zhu, X. Yang, M. Huang, J. Huang, D. Liang, X. Zhu, D. Li, A. Pan, *Nano Research* **2022**, 15, 3539–3547.

[2] M. M. Islam, D. Dev, A. Krishnaprasad, L. Tetard, T. Roy, Sci. Rep. 2020, 10, 21870.

[3] T. Ahmed, S. Kuriakose, E. L. H. Mayes, R. Ramanathan, V. Bansal, M. Bhaskaran, S. Sriram, S. Walia, *Small* **2019**, 15, 22.

[4] R. A. John, F. Liu, N. A. Chien, M. R. Kulkarni, C. Zhu, Q. Fu, A. Basu, Z. Liu, N. Mathews, *Advanced Materials* **2018**, 30, 25.

[5] W. Wang, S. Gao, Y. Li, W. Yue, H. Kan, C. Zhang, Z. Lou, L. Wang, G. Shen, *Advanced Functional Materials* **2021**, 31, 34.

[6] Y. Wang, J. Yang, Z. Wang, J. Chen, Q. Yang, Z. Lv, Y. Zhou, Y. Zhai, Z. Li, S. T. Han, *Small* **2019**, 15, 7.

[7] F. W. Shuchao Qin, Yujie Liu, Qing Wan, Xinran Wang,Yongbing Xu,Yi Shi, Xiaomu Wang, a. R. Zhang, *Small* **2017**, 4, 3.

[8] Y. Wang, Z. Lv, J. Chen, Z. Wang, Y. Zhou, L. Zhou, X. Chen, S. T. Han, *Advanced Materials* **2018**, 30, 38.

[9] S. Dai, X. Wu, D. Liu, Y. Chu, K. Wang, B. Yang, J. Huang, *ACS Applied*

Materials & Interfaces **2018**, 10, 25.
[10] K. Wang, S. Dai, Y. Zhao, Y. Wang, C. Liu, J. Huang, *Small* **2019**, 15, 11.
[11] N. Duan, Y. Li, H. C. Chiang, J. Chen, W. Q. Pan, Y. X. Zhou, Y. C. Chien, Y. H. He, K. H. Xue, G. Liu, T. C. Chang, X. S. Miao, *Nanoscale* **2019**, 11, 38.
[12] Y. Sun, L. Qian, D. Xie, Y. Lin, M. Sun, W. Li, L. Ding, T. Ren, T. Palacios, *Advanced Functional Materials* **2019**, 29, 28.
[13] M. Lee, W. Lee, S. Choi, J. W. Jo, J. Kim, S. K. Park, Y. H. Kim, *Advanced Materials* **2017**, 29, 28.
[14] L. Yin, W. Huang, R. Xiao, W. Peng, Y. Zhu, Y. Zhang, X. Pi, D. Yang, *Nano Letters* **2020**, 20, 5.
